# Consensus recommendations for management of patients with type 2 diabetes mellitus and cardiovascular diseases

**DOI:** 10.1186/s13098-019-0476-0

**Published:** 2019-09-26

**Authors:** Alaaeldin Bashier, Azza Bin Hussain, Elamin Abdelgadir, Fatheya Alawadi, Hani Sabbour, Robert Chilton

**Affiliations:** 1 Department of Endocrinology, Dubai Health Authority, Dubai Hospital, P.O. Box 94132, Dubai, UAE; 2 Cleveland Clinic Abu Dhabi, Heart and Vascular Institute, Al Maryah Island, Abu Dhabi, UAE; 3 Division of Cardiology, University of Texas Health Science Center, Audie L Murphy VA Hospital, San Antonio, TX USA

**Keywords:** T2DM, Consensus, CVOTs, CVDs, HF

## Abstract

The recent American Diabetes Association and the European Association for the Study of Diabetes guideline mentioned glycaemia management in type 2 diabetes mellitus (T2DM) patients with cardiovascular diseases (CVDs); however, it did not cover the treatment approaches for patients with T2DM having a high risk of CVD, and treatment and screening approaches for CVDs in patients with concomitant T2DM. This consensus guideline undertakes the data obtained from all the cardiovascular outcome trials (CVOTs) to propose approaches for the T2DM management in presence of CV comorbidities. For patients at high risk of CVD, metformin is the drug of choice to manage the T2DM to achieve a patient specific HbA1c target. In case of established CVD, a combination of glucagon-like peptide-1 receptor agonist with proven CV benefits is recommended along with metformin, while for chronic kidney disease or heart failure, a sodium–glucose transporter proteins-2 inhibitor with proven benefit is advised. This document also summarises various screening and investigational approaches for the major CV events with their accuracy and specificity along with the treatment guidance to assist the healthcare professionals in selecting the best management strategies for every individual. Since lifestyle modification and management plays an important role in maintaining the effectiveness of the pharmacological therapies, authors of this consensus recommendation have also briefed on the patient-centric non-pharmacological management of T2DM and CVD.

## Background and rationale

The global incidence and prevalence of type 2 diabetes mellitus (T2DM) has quadrupled since 1980 and still escalating [1]. Cardiovascular diseases (CVDs)-majorly coronary artery disease (CAD), heart failure (HF), and stroke are the major cause of death and disability in patients with T2DM [2, 3]. An area where attention in particular is needed is patients with co-existing T2DM and HF. A recent data showed alarming progression in the risk of cardiovascular (CV) death and hospitalization for heart failure (HHF) in patients with heart failure (HF) and T2DM, compared to those with HF without T2DM [4]. Although improved evidence-based treatment has led to improved survival, the 5-year mortality rate in the patients with advanced HF is approximately 50% [5, 6], and in some regions, the number of deaths from HF has surpassed the number of deaths from myocardial infarction (MI) in T2DM patients [7].

Due to the clinical burden of CVD complications observed in T2DM patients, the awareness on the joint management of T2DM and CVD has been increased. Though the significance of intensive glycemic control for protection against microvascular complications and CVD in people with type 1 diabetes mellitus is well established [8, 9], its role for reducing CV risk has not been established as clearly in people with T2DM [10–12].

As a result, there is an increasing pressure from regulatory agencies that the anti-hyperglycaemic agents (AHAs) should demonstrate CV safety and benefits in T2DM patients, especially for major CV events such as CV mortality, HF, and non-fatal MI [13, 14]. Following these regulatory requirements, several CV outcomes trials (CVOTs) have been carried out that assessed the CV safety of the AHAs. These CVOTs indicated comparatively lower risk of CVDs associated with certain agents as compared to the others [15–18]. This has triggered a major paradigm shift beyond glucose control, to a broader strategy of comprehensive CV risk reduction [19].

In majority of patients with concomitant T2DM and CVD, the presence of certain comorbidities (e.g. atherosclerotic CVD, HF, chronic kidney disease, obesity) mandate a specific approach to the choice of glucose-lowering agents. This document, therefore, proposes an approach for the management of glycaemia in patients with T2DM and the above-mentioned comorbidities. It also elaborates on screening as well as management of major CV events in patients with concomitant T2DM, and prevention of these complications by lifestyle modifications in form of patient-centred care. This consensus recommendation helps health professionals to make decisions in their daily practice. However, the final decisions concerning every patient must be made by the responsible health professionals through a detailed consultation with the patient and the caregiver.

## Methodology

The consensus is comprised of three major parts: (1) risk stratification and treatment of T2DM in patients with corresponding risk/history of CVD, (2) risk stratification, screening as well as treatment of CVD in patients with comorbidity of T2DM, (3) discussion on lifestyle management for this special set of population. The literature search of the current evidence was performed using the MEDLINE (by PubMed), Cochrane, and Google scholar databases. The articles included randomized control studies, cohort or case–control studies, systematic reviews, meta-analyses, prospective and retrospective studies, clinical practice guidelines, and evidence-based consensus recommendations/guidelines. All articles included were in English language. The rationale for prioritizing the treatment approach in case of patients T2DM and CVD was based on the evidence grading system used by the American Diabetes Association [20] and American College of Cardiology/American Heart Association task force [21]. The level of evidences was categorised as follows:

- Level 1: evidence from randomized control studies, systematic reviews and meta-analyses.
- Level 2: evidence from comparative, case–control and descriptive studies.
- Level 3: evidence from non-randomized, prospective or retrospective studies.
- Level 4: expert committee reports or clinical opinions of respected authorities.

A multidisciplinary expert panel of specialised endocrinologists and cardiologists, with clinical and research expertise in the diagnosis and treatment of T2DM and CVD, was convened and recommendations were formulated. All the available evidences were comprehensively reviewed, discussed, developed, and final decisions were made by the panel. Based on the above-mentioned level of evidences, recommendations were graded as follows:

- Grade A: based on evidence Level 1.
- Grade B: based on evidence Level 2 or extrapolated from Level 1.
- Grade C: based on evidence Level 3 or extrapolated from Level 1 and 2.
- Grade D: based on evidence Level 4 or extrapolated from Levels 1–3.

### Clinical terms used in the consensus recommendation [11, 15, 16, 22–36]

#### ASCVD

Atherosclerotic cardiovascular disease (ASCVD) is defined somewhat differently across trials; however, all the CVOTs overall had similar inclusion criteria. These criteria generally included a history of any of the conditions mentioned hereafter: acute coronary syndrome or MI, stable or unstable angina, CAD with or without revascularization, any other arterial revascularization, peripheral artery disease, stroke assumed to be atherosclerotic in origin. The relevant conditions compatible with clinically significant atherosclerosis consisted of transient ischaemic attack, hospitalised for unstable angina, amputation, congestive heart failure New York Heart Association class II–III, arterial stenosis of more than 50%, symptomatic/asymptomatic CAD documented by imaging, chronic kidney disease (CKD) with estimated GFR (eGFR) < 60 mL/min/1.73 m^2^. Certain trials had included few patients without clinical ASCVD but required them to have a high burden of risk factors based on age and the presence of two or more cardiac risk factors.

#### MACE

In this document, three major adverse cardiovascular events (MACE) are considered as primary endpoints in the CVOTs [37]:

- 3-point MACE: composite of non-fatal MI, non-fatal stroke, or cardiovascular mortality.
- 4-point MACE: 3-point MACE + hospitalisation for unstable angina.
- 5-point MACE: 3-point MACE + hospitalisation for heart failure (HF) or unstable angina.

#### High risk of CVD

Patients with high risk for CVD included multiple risk factors for CV disease: men 55 years of age or older or women 60 years of age or older in addition to another traditional risk factor like hypertension, dyslipidemia (defined as a low-density lipoprotein cholesterol level > 130 mg/dL [3.36 mmol/L]) or the use of lipid-lowering therapies, and smoking. Furthermore, microalbuminuria or macroalbuminuria [urinary albumin:creatinine ratio (UACR) higher than 30 mg/g or equivalent]; high renal risk, which included (a) eGFR of 45–75 mL/min/1.73 m^2^ and UACR higher than 200 mg/g or equivalent or (b) eGFR of 15–45 mL/min/1.73 m^2^ regardless of UACR. Participants with end-stage renal disease (eGFR less than 15 mL/min/1.73 m^2^) or requiring maintenance dialysis, history of CAD; stroke or peripheral vascular disease were excluded [38].

### Anti-hyperglycaemic agents (AHAs) and related cardiovascular outcome trials

The most common classes of AHAs, their mechanism of actions, adverse effects, and associated CV favourable/neutral/unfavourable outcomes are summarized in Table 1.

**Table 1:** Summary of commonly used anti-hyperglycaemic agents

Drug class	Anti-hyperglycaemic agents	Estimated HbA1c reduction (%)	Route of administration	Mechanism of action	Impact on CV events	Advantages	Disadvantages	Contraindications	Comments	CV favourability
Biguanide	Metformin	1	Oral	Activates AMPK	Reduction in MI, all-cause mortality	Extensive experience, no hypoglycaemia, inexpensive	Diarrhoea, nausea, GI symptoms, vitamin B12 deficiency, lactic acidosis (rare)	Acidosis, severe CHF, hypovolaemia, if intravenous contrast to be used, hold on the day of study and restart 48 h after the contrast if eGFR > 30 mL/min/1.73 m^2^	Modest weight loss, reduced CV event rates, caution to be exercised or dose adjustment for CKD stage 3B (eGFR 30–44 mL/min/1.73 m^2^)	Favourable
GLP-1 receptor agonist	Liraglutide, semaglutide, exenatide, lixisenatide, dulaglutide, albiglutide	0.8–1.5	Injectable	Activate GLP-1 receptor, ↑ insulin secretion, ↓ glucagon secretion	Reduction in CV mortality, all-cause mortality, MI/stroke (liraglutide, semaglutide, Dulaglutide)	No hypoglycaemia as monotherapy, ↓ weight, excellent postprandial glucose efficacy for meals after injections, improves CV risk factors (liraglutide, semaglutide, Dulaglutide)	Higher rates of retinopathy with semaglutide, frequent and transient GI side effects, modestly ↑ heart rate, acute pancreatitis (rare/uncertain), very high cost	History of pancreatitis, personal or family history of medullary thyroid cancer or multiple endocrine neoplasia 2, not to be used with DPP4 inhibitors		Favourable
DPP-4 inhibitor	Sitagliptin, linagliptin, saxagliptin, alogliptin	0.6–0.8	Oral	Prevent degradation of GLP-1	Increased HF hospitalization (saxagliptin)	No hypoglycaemia, weight neutral, well tolerated	Nausea (generally resolves), upper respiratory tract complaints	Rare urticaria/angioedema, pancreatitis^a^, arthralgia^a^, bullous pemphigoid^a^	No increase in CV risk (except hospitalisation for HF) compared to other agents in high risk patients (SAVOR-TIMI53), dose adjustment/avoidance for renal disease depending on agent (except for Linagliptin)	Neutral (exception: saxagliptin–unfavourable)
SGLT2 inhibitors	Canagliflozin, dapagliflozin, empagliflozin	0.5–0.6	Oral	Block glucose reabsorption in proximal renal tubule	Reduction in CV mortality only with empagliflozin (EMPA-REG), reduction in HF hospitalization with empagliflozin (EMPA-REG), canagliflozin (CANVAS) and dapagliflozin (DECLARE-TIMI 58)	No hypoglycaemia, ↓ weight, ↓ blood pressure, effective at all stages of T2DM with preserved glomerular function, ↓ MACE, CKD with some agents	GU infections, polyuria, hypovolaemia/hypotension/dizziness, ↑ LDL-C, ↑ creatinine (transient), euglycaemic ketoacidosis (rare), Fournier’s gangrene (very rare), expensive, canagliflozin: increased risk for amputation [canagliflozin (0.6% vs. 0.3% in placebo)], bone fracture, severe PVD, neuropathy, and DFU. No increased risk of amputation seen for empagliflozin or dapagliflozin to date	Severe renal impairment, ESRD or dialysis	Dose adjustment/avoidance for renal disease, use lower doses of canagliflozin and empagliflozin if eGFR < 60 mL/min/1.73 m^2^	Favourable
Thiazolidinedione	Pioglitazone	0.5–1.4	Oral	Bind PPAR-γ, decrease insulin resistance and increase glucose utilization	Increased risk of HF; pioglitazone associated with reduced MACE	Low risk for hypoglycaemia, durability, ↑ HDL-C, ↓ triacylglycerol (pioglitazone), ↓ ASCVD events (pioglitazone: in a post-stroke insulin- resistant population and as a secondary endpoint in a high-CVD-risk diabetes population), lower cost	Weight gain, peripheral oedema/HF in patients with underlying disease, bone loss, ↑ bone fractures, ↑ LDL-C, bladder cancer^a^, macular oedema^a^	Severe heart disease at risk for CHF, NYHA Class III or IV HF, liver disease	Increased risk of fluid retention. Pioglitazone is neutral to beneficial for composite CV outcomes (PROactive)	Favourable for MACE but increased risk of HF
α-Glucosidase Inhibitor	Acarbose, miglitol	0.5–1.0	Oral	Reduces absorption of dietary carbohydrate	Improve the CV risk factors	↓ postprandial glucose excursions, non-systemic mechanism of action, CV safety, lower cost	GI discomfort, flatulence, diarrhoea, elevated transaminases, frequent GI side effects, frequent dosing schedule	Chronic intestinal disorders, moderate to severe renal impairment (creatinine > 2 mg/dL), caution in cirrhosis	May reduce CV risk in patients with impaired glucose tolerance, dose adjustment/avoidance for renal disease	Favourable
Basal insulins (long acting)	Degludec, glargine	1.0–1.7	Injectable	Activate insulin receptor, ↓ glucose production	Neutral CV effects	Nearly universal response, once daily injection	Hypoglycaemia, weight gain, training requirements, frequent dose adjustment for optimal efficacy, high cost	Not reported	Severe hypoglycaemia may increase the risk of death for up to 1 year after occurrence	Neutral

*AMPK* 5ʹ adenosine monophosphate-activated protein kinase, *ASCVD* atherosclerotic cardiovascular disease, *CHF* congestive heart failure, *CKD* chronic kidney disease, *CVD* cardiovascular disease, *eGFR* estimated glomerular filtration rate, *ESRD* end-stage renal disease, *GI* gastrointestinal, *GU* genitourinary, *HDL/LDL* high density lipoprotein/low density lipoprotein, *HF* heart failure, *MACE* major adverse cardiovascular event, *DFU* diabetic foot ulcer, *NYHA* New York Heart Association, *PVD* peripheral vascular disease, *T2DM* type 2 diabetes mellitus, *DPP-4* dipeptidyl peptidase 4, *GLP-1* glucagon like peptide 1, *PPAR* peroxisome proliferator-activated receptor, *SGLT-2* sodium–glucose cotransporter 2. Full names of all the cardiovascular outcome trials stated in this table have been mentioned in the ‘abbreviations’ section of the manuscript

^a^Incidence rate under observation

Until recently, there were no AHAs robustly proven safe or effective with regard to CV outcomes. In 2008, the Food and Drug Administration and European Medicines Agency mandated formal evaluation of CV safety of all new AHAs for the treatment of T2DM [39] therefore, results from numerous randomized controlled trials projecting the CV safety profiles of AHAs are now available. These agents, through the CVOTs, have demonstrated not only CV safety but also significant CV benefit for selected therapies. The key points of the CVOTs are summarized in the Table 2.

**Table 2:** Summary of cardiovascular outcome trials

Drug class	Trial/drug	Inclusion criteria	Prior CVD/CHF (%)	No. of patients	Primary endpoint [HR (95% CI)]	Key secondary endpoints [HR (95% CI)]					
					Relative risk reduction (%)	Relative risk reduction (%)					
						Unstable angina hospitalisation	Stroke^d^	MI^d^	CV death	HF hospitalisation	All-cause mortality
Biguanide	UKPDS/metformin	Newly diagnosed T2DM patients aged 25–65 years and had a fasting plasma glucose > 6 mmol/L on two mornings, 1–3 weeks apart	NR	1704	0.68 (0.53–0.87)	NR	0.59 (0.29–1.18)	0.61 (0.41–0.69)	0.58 (0.37–0.91)	0.79 (0.27–1.07)	0.64 (0.45–0.91)
								39			24
DPP4 inhibitors	SAVOR TIMI 5325/saxagliptin	T2DM and history of or multiple risk factors for CVD	78/13	16,492	3-point MACE 1.00 (0.89–1.12)	1.27 (1.07–1.51)	1.11 (0.88–1.39)	0.95 (0.80–1.12)	1.03 (0.87–1.22)	1.27 (1.07–1.51)	1.11 (0.96–1.27)
	CARMELINA/linagliptin	T2DM and high CV risk [history of vascular disease and urine-albumin creatinine ratio > 200 mg/g)], and high renal risk (reduced eGFR and micro- or macroalbuminuria)	58/46	6991	3-point MACE 1.02 (0.89–1.17)	0.87 (0.57–1.31)	0.88 (0.63–1.23)	1.15 (0.91–1.45)	0.96 (0.81–1.14)	0.90 (0.74–1.08)	0.98 (0.84–1.13)
	EXAMINE/alogliptin	T2DM and ACS within 15–90 days before randomization	100/28	5380	3-point MACE 0.96 (≤ 1.16)*	0.90 (0.60–1.37)	0.91 (0.55–1.50)	1.08 (0.88–1.33)	0.85 (0.66–1.10)	1.19 (0.90–1.58)	0.88 (0.71–1.09)
	TECOS/sitagliptin	T2DM and pre-existing CVD	74/18	14,671	4-point MACE 0.98 (0.88–1.09)	0.90 (0.70–1.16)	0.97 (0.79–1.19)	0.95 (0.81–1.11)	1.03 (0.89–1.19)	1.00 (0.83–1.20)	1.01 (0.90–1.14)
SGLT2 inhibitor	EMPA-REG OUTCOME/empagliflozin	T2DM and pre-existing CVD, with BMI ≤ 45 kg/m^2^ and eGFR ≥ 30 mL/min/1.73 m^2^	99/10	7020	3-point MACE 0.86 (0.74–0.99)	0.99 (0.74–1.34)	1.18 (0.89–1.56)	0.87 (0.70–1.09)	0.62 (0.49–0.77)	0.65 (0.50–0.85)	0.68 (0.57–0.82)
					14		24	13	38	35	32
	Integrated CANVAS programme (CANVAS, CANVAS-R)/canagliflozin	T2DM and pre-existing CVD at ≥ 30 years of age or ≥ 2 CV risk factors (T2DM ≥ 10 years, systolic blood pressure > 140 mmHg and on anti-hypertensive agents, current smoking, micro- or macroalbuminuria) at ≥ 50 years of age	65.6/14.4	10,142	3-point MACE 0.86 (0.75–0.97)^b^	NR	0.87 (0.69–1.09)^b^	0.89 (0.73–1.09)^b^	0.96 (0.77–1.18)^c^, 0.87 (0.72–1.06)^b^	0.67 (0.52–0.87)^b^	0.87 (0.74–1.01), 0.90 (0.76–1.07)^b^
					14		10	15	13	33	10
	DECLARE-TIMI 58/dapagliflozin	T2DM with creatinine clearance of ≥ 60 mL/min, eGFR < 60 mL/min/1.73 m^2^, had multiple risk factors for ASCVD or had established ASCVD (clinically evident ischemic heart disease, ischemic cerebrovascular disease, or peripheral artery disease)	41/10	17,160	Total cohort, 0.93 (0.84–1.03); ASCVD, 0.90 (0.79–1.02); multiple risk factors without ASCVD, 1.01 (0.86–1.20)	NR	1.01 (0.84–1.21)	0.89 (0.77–1.01)	Total cohort, 0.98 (0.82–1.17); ASCVD, 0.83 (0.71–0.98); multiple risk factors without ASCVD, 0.84 (0.67–1.04)	Total cohort, 0.73 (0.61–0.88); ASCVD, 0.83 (0.71–0.98); multiple risk factors without ASCVD, 0.84 (0.67–1.04)	0.93 (0.82–1.04)
GLP1-receptor agonist	LEADER/liraglutide	T2DM and pre-existing CVD, kidney disease, or HF at ≥ 50 years of age or ≥ 1 CV risk factor at ≥ 60 years of age (microalbuminuria or proteinuria, hypertension and left ventricular hypertrophy, left ventricular systolic or diastolic dysfunction)	81/18	9340	3-point MACE 0.87 (0.78–0.97)	0.98 (0.76–1.26)	0.86 (0.71–1.06)	0.86 (0.73–1.00)	0.78 (0.66–0.93)	0.87 (0.73–1.05)	0.85 (0.74–0.97)
					13		11	12	22	13	15
	SUSTAIN-6/semaglutide	T2DM and pre-existing CVD, HF, or CKD at ≥ 50 years	60/24	3297	3-point MACE 0.74 (0.58–0.95)	0.82 (0.47–1.44)	0.61 (0.38–0.99)	0.74 (0.51–1.08)	0.98 (0.65–1.48)	1.11 (0.77–1.61)	1.05 (0.74–1.50)
					6		39	26	2	11	5
	ELIXA/lixisenatide	T2DM and an acute coronary event within 180 days before screening	100/22	6068	4-point MACE 1.02 (0.89–1.17)	1.11 (0.47–2.62)	1.12 (0.79–1.58)	1.03 (0.87–1.22)	0.98 (0.78–1.22)	0.96 (0.75–1.23)	0.94 (0.78–1.13)
	EXSCEL/exenatide	T2DM with or without pre-existing CVD	73.1/16.2	14,752	3-point MACE 0.91 (0.83–1.00)	1.05 (0.94–1.18)	0.85 (0.70–1.03)	0.97 (0.85–1.10)	0.88 (0.76–1.02)	0.94 (0.78–1.13)	0.86 (0.77–0.97)
	Harmony/albiglutide	T2DM and established disease of MI, ≥ 50% carotid artery stenosis/peripheral artery circulation at ≥ 40 years	87/–	9463	3-point MACE 0.78 (0.68–0.90)	NR	NR	0.75 (0.61–0.90)	0.93 (0.73–1.19)	0.85 (0.70–1.04) (composite of CV death and HF hospitalization)	0.95 (0.79–1.16)
Thiazolidinediones	IRIS/pioglitazone	Insulin resistance but not diabetes + ischemic stroke or TIA in 6 months before randomization	100/–	3876	Composite of fatal and nonfatal stroke and MI 0.76 (0.62–0.93)	NR	0.82 (0.61–1.10)	NR	NR	–	0.93 (0.73–1.17)
							18				20
	PROactive/pioglitazone	T2DM with history of pre-existing macrovascular disease (MI, stroke, percutaneous coronary intervention or coronary artery bypass surgery ≥ 6 months of study, ACS ≥ 3 months before study entry, or objective evidence of coronary artery disease or obstructive arterial disease in the leg) making the patient population at very high-risk for macrovascular disease	100/–	5238	Composite MACE 0.90 (0.80–1.02)^a^	NR	0.80 (0.72–0.98) (composite of all-cause mortality, MI, stroke)	0.80 (0.72–0.98)	0.90 (0.80–1.02)	NR	0.80 (0.72–0.98)
					10		16	16			16
Insulin	ORIGIN/insulin glargine	T2DM with CV risk factors plus impaired fasting glucose, impaired glucose tolerance, or type 2 diabetes to receive insulin glargine	59/–	12,537	1.02 (0.94–1.11)	0.91 (0.76–1.08)	1.03 (0.89–1.21)	1.02 (0.88–1.19)	1.00 (0.89–1.13)	0.90 (0.77–1.05)	0.98 (0.90–1.08)
	DEVOTE/degludec	T2DM, age > 50 years + history of CVD and/or CKD, age > 60 years + > 1 CV risk factors	83/–	7637	3-point MACE 0.91 (0.78–1.06)	0.95 (0.68–1.31)	0.90 (0.65–1.23)	0.85 (0.68–1.06)	0.96 (0.76–1.21)	NR	0.91 (0.76–1.11)
α-glucosidase inhibitors	ACE/acarbose	Coronary heart disease and impaired glucose tolerance (conducted in China)	100/3.7	6522	5-point MACE 0.98 (0.86–1.11)	1.02 (0.82–1.26)	0.97 (0.70–1.33)	1.12 (0.87–1.46)	0.89 (0.71–1.11)	0.89 (0.63–1.24)	0.98 (0.81–1.19)

*ACS* acute coronary syndrome, *ASCVD* atherosclerotic cardiovascular disease, *MACE* major adverse cardiovascular event, *CKD* chronic kidney disease, *CVD* cardiovascular disease, *MI* myocardial infarction, *HF* heart failure, *HR* hazard ratio, *TIA* transient ischaemic attack, *T2DM* type 2 diabetes mellitus, *DPP4* dipeptidylpeptidase-4, *GLP1* glucagon like peptide 1, *NR* not reported, *SGLT2i* sodium–glucose cotransporter 2 inhibitor. Full names of all the cardiovascular outcome trials have been mentioned in the ‘abbreviations’ section of the manuscript. *Primary endpoints*: 3-point MACE: CV death, non-fatal MI, non-fatal stroke, 4-point MACE: 3-point MACE + hospitalisation for unstable angina, 5-point MACE: 3-point MACE + hospitalization for HF or unstable angina, – not available

^a^Composite MACE (PROactive trial): CV death, non-fatal MI (including silent MI), stroke acute coronary syndrome, coronary or leg artery revascularization, or above the ankle amputation

^b^Non-truncated integrated data (refers to pooled data from CANVAS, including before 20 November 2012 plus CANVAS-R)

^c^Truncated integrated data set (refers to pooled data from CANVAS after 20 November 2012 plus CANVAS-R; pre-specified in treating hierarchy as the principal data set for analysis for superiority of all-cause mortality and CV death in the CANVAS Program)

^d^Reported for fatal and nonfatal events in all trials except EXAMINE, ELIXA, and SUSTAIN-6, which reported for nonfatal events only

The CVOTs were mainly designed to rule out unacceptable CV risk, but some were powered to estimate superiority after non-inferiority was demonstrated. Researchers have typically studied the T2DM population in which some or all individuals had advanced risk/history of ASCVD or established CVD to ensure the accrual of sufficient events in a timely manner (such as the presence or absence of ASCVD, HF with CKD, and more such conditions affecting the population with T2DM).

### Consensus recommendations for the management of T2DM and CVD through lifestyle management

Type 2 diabetes is related to adverse lifestyle, hence any intervention to improve outcomes in type 2 diabetes should start with therapeutic life style changes.

#### Recommendation: we recommend modifying the dietary habits and adapting the novel proven dietary interventions like medical nutritional therapy for the management of T2DM and CVD (Grade A) [40, 41]

The Look AHEAD (Action for Health in Diabetes) trial [42] was one of the major trials that evaluated benefits of lifestyle intervention. The study proved no direct benefits of intense lifestyle interventions (dietary restrictions, weight loss, etc.) on the CV mortality. The benefits observed with this study were mostly on glycemic control.

For a strict control of T2DM and its subsequent impact on the CV risk, it is important to look into the glycaemic index (GI = blood glucose response 2 h after intake of 100 g of food ÷ blood glucose response on intake of 100 g glucose) and glycaemic load (GL = carbohydrate content of the item × its GI ÷ 100) of an individual food item. Multiple studies have concluded the beneficial effects of diet with low GI (< 55) on the improvement of T2DM and its complications. Diets with low GL (≤ 10) have also shown benefits in managing T2DM and reducing complications in several studies. Patients with CVDs are advised to reduce the saturated fats intake and improve the mono-unsaturated fat intake to prevent or ameliorate further worsening of the condition [43–46].

The novel dietary interventions such as medical nutritional therapy (MNT) mainly target management of blood glucose and CV risk factors by reducing the risk for diabetes-related complications and optimal co-ordination of dietary intake with pharmacological therapies to achieve a favourable outcome. Two basic aspects of MNT include dietary quality and energy restriction. Strategies directed at both dimensions can improve glycaemic control.

MNT not only aims to control blood glucose, but also to improve other co-morbidities such as dyslipidaemia, obesity, and hypertension. MNT can be utilised as a primary, secondary, or tertiary prevention measure in T2DM. Primary prevention measures of MNT are by modifying diets in high-risk individuals (such as pre-diabetes, central obesity etc.) to prolong or prevent the onset of T2DM. Secondary prevention measures aim to achieve tight glycaemic control by dietary modification; in turn, reducing diabetic complications in patients with T2DM. Tertiary prevention measures are to manage diabetes-related complications such as CVDs or renal diseases in those with T2DM. Likewise, patients at risk or history of CVD can also prevent further consequences by modifying diet to one of the four diet patterns that have been evidently proven to be effective in such cases: low fat diet, low carbohydrate diet, Mediterranean diet, and DASH diet (Dietary Approach to Stop Hypertension diet).

#### Recommendation: we recommend maintaining physical fitness as well as physical activity as an integral part of T2DM management in patients with concomitant CVD (Grade A, B) [40, 41, 47]

Physical activity improves insulin sensitivity, body weight, CV risk factors, physical fitness, lipid level, blood pressure, overall well-being, and also reduces the risk of CV morbidity and mortality. It improves the adverse lipid profile by lowering the total cholesterol as well as low-density lipoprotein cholesterol and increasing the high-density lipoprotein cholesterol. These, in turn, reduce the risk of various CV events inherent to patients with T2DM.

Healthcare providers should evaluate and examine the patients with T2DM before starting an exercise programme, especially individuals leading sedentary lifestyles and at risk/history of CVDs. In case of overweight or obese patients with high risk/history of CVD, one needs to opt for individual-specific support and care. Furthermore, providers should look for the conditions (uncontrolled diabetes, uncontrolled hypertension, HF, diabetic peripheral neuropathy, etc.) which are either contraindicated or lead to increased risk of morbidity with certain types of exercise.

### Miscellaneous management (Grade B)

Sleep deprivation and poor quality of sleep cause decline in the metabolic and hormonal function, leading to the development of T2DM and CVD. It is advised to have 6 to 8 h of uninterrupted sleep at night [40]. This also applies to managing the stress level, adults with T2DM and CVD should take several measures such as meditation or yoga to avoid stress. The CVD patients are further advised to avoid smoking and avoid/limit alcohol as well as caffeine consumption [48].

Overall, the most effective approach for the prevention of macrovascular complications in T2DM and CVD appears to be multifactorial risk reduction (glycaemic control, smoking cessation, diet, exercise, aggressive blood pressure control, dyslipidemia management). This, in conjunction with pharmacological therapies, would help the patients lead the life with lesser disease burden.

## Consensus recommendations for the management of T2DM in patients with corresponding high risk/history of CVD

The results of all the below mentioned CVOTs are summarized in Table 2. Based on these CVOTs and years of expertise, the authors have come up with the treatment approaches for T2DM patients with high risk/history of CVDs.

### Management of T2DM in patients at a risk of CVD

**Fig. 1:**
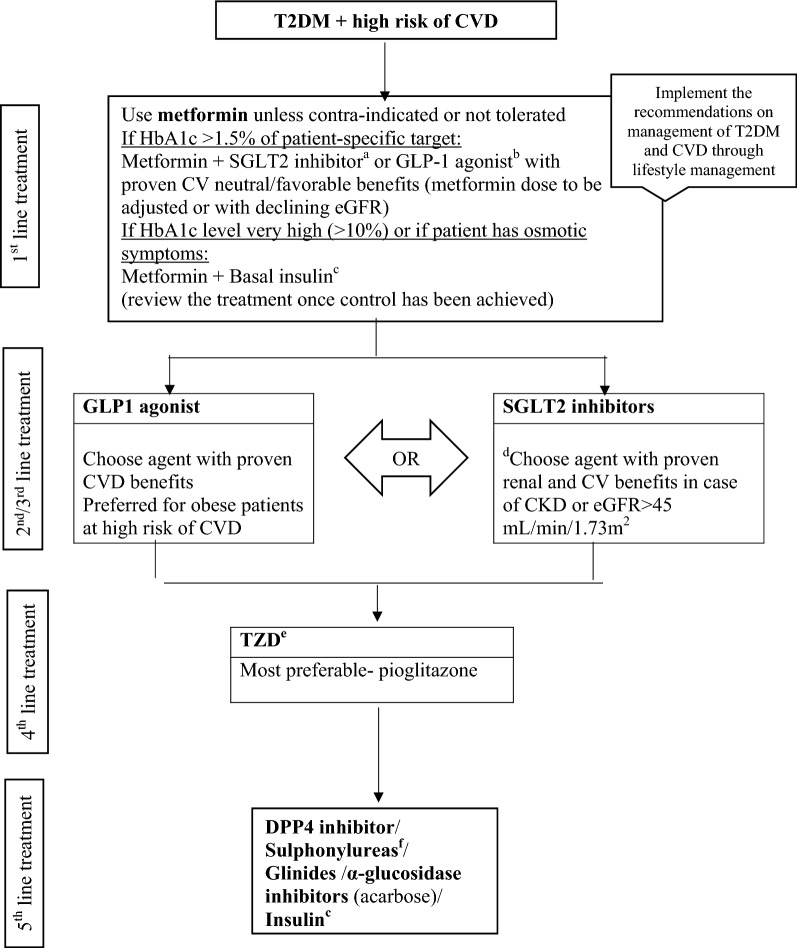
Evidence-based algorithm for the management of patients with T2DM and high risk of CVD. *CVD* cardiovascular disease, *GLP-1* glucagon-like peptide-1, *SGLT2* sodium–glucose transporter proteins-2, *TZD* thiazolidinedione, *DPP4* dipeptidylpeptidase-4. ^a^Proven CVD benefits means the agent has a label indication of reducing the CVD events. For SGLT2 inhibitors evidence based preference is empagliflozin > canagliflozin. SGLT2 inhibitors vary in regards to eGFR pre-requisites for a continued use. ^b^For GLP-1 agonist evidence based preference is Semaglutide > Liraglutide > Dulaglutide > Exenatide > Lixenatide. Caution to be exercised in case of end-stage renal disease. ^c^Degludec and insulin Glargine (U100) have shown CVD safety, ^d^Dapagliflozin: preferred option for patients with eGFR > 60 mL/min/1.73 m^2^, ^e^Low dose TZDs are better tolerated. ^f^Choose later generation SU to minimize the risk of hypoglycaemia

Figure 1 describes the consensus approach to glucose lowering with AHAs in this set of population. For patients not reaching their target HbA1c, it is advisable to assess adherence and arrange timely follow-up every 3 months.

#### Recommendation: we recommend using metformin alone or in combination with other drugs as first line therapy in patients with T2DM and high risk of CVD (Grade A)

One of the earliest CVOTs in T2DM patients, UKPDS 34, determined whether a policy of reducing hyperglycaemia to near-normal levels will reduce the risk of development or progression of diabetes-related complications. Metformin was among the intensive control group, which demonstrated statistically significant reductions in the risk of MI and CV death [11]. This rendered metformin a drug of choice in the view of previously available insulin and sulphonylurea that had risk of hypoglycaemia and weight gain. With a long-standing experience and global use of metformin, the Food and Drug Administration removed boxed warnings contraindicating its use in patients with HF in 2006, and liberalized contraindications for those with kidney disease in 2016 [49].

After UKPDS, all the CVOTs were on top of metformin as baseline therefore, the researchers consider the CV benefits of these trials inseparable from the metformin, making it a foundation for their effects (see Table 2) [50]. An interesting analysis of 24,752 patients with T2DM carried out by Boussageon et al. [51] showed lesser CV events if the HbA1c was brought down to 6.5% early during the initial 6 months from the time of metformin initiation. Interestingly, this study showed a J-shape pattern of CV events with HbA1c extremes, patients with higher HbA1c at 6 months had 30% higher incidences of CV events (MI, stroke, CV mortality). A novel observation in this study was- the more HbA1c reduction attained in the initial 6 months from metformin initiation, the lower long-term CV events.

It is important to note that recent meta-analyses challenged the CV benefit of metformin and concluded that it could provide CV safety but not efficacy [52, 53]. Nonetheless, the non-metformin users in the latest CVOTs ranged between 18 and 40%, which further gives a significant power to claim that CVD prevention was attained solely by the newer AHAs along with a robust CV safety and efficacy evidence. Despite of this, metformin is still recommended as a first line treatment for patients with T2DM, even for those with prevalent ASCVD, and is the most-prescribed AHA worldwide [11, 47]. Also, in the EMPA-REG OUTCOME trial [26], a sub-group analysis suggested that baseline metformin use helped patients in exaggerated effect of empagliflozin on CV events during the trial {not on metformin: [hazard ratio (HR): 0.72; 95% confidence interval (CI) 0.56–0.94; p < 0.0001], on metformin: (HR: 0.92; 95% CI 0.77–1.10); p = 0.14}. Furthermore, metformin is the only AHA to show reduced macrovascular risk in overweight T2DM, and it remains the first-line agent of choice recommended by most treatment guidelines [53].

#### Recommendation: we recommend starting GLP1-receptor agonists (GLP1-RA) or SGLT2 inhibitors (SGLT2i) as a second/third line therapy in patients with T2DM and who are at high risk for CVD or renal impairment (Grade A)

Patients at high risk of CVD or those with CKD and have eGFR > 45 mL/min/1.73 m^2^ are recommended to choose SGLT2i with proven primary renal and CV benefits as second line therapy.

Firstly, in case of T2DM patients with obesity and high risk of CVD, a GLP1-RA might be a logical as a second line therapy. Argument might arise on the availability of evidence for GLP1-RA in primary prevention, as CV benefits in LEADER, SUSTAIN-6, and HARMONY trials were seen in patients with established ASCVD [15, 16, 29]. However, data from the REWIND trial (Researching Cardiovascular Events with a Weekly Incretin in Diabetes) that assessed the CVOT of dulaglutide in high risk patients showed statistically significant reduction in primary CV outcome. the primary composite outcome occurred in 594 (12.0%) participants at an incidence rate of 2.4 per 100 person-years in the dulaglutide group and in 663 (13.4%) participants at an incidence rate of 2.7 per 100 person-years in the placebo group (hazard ratio [HR] 0.88, 95% CI 0.79–0.99; p = 0.026) [54]. It is essential to mention that benefits were mainly driven by reduction in stroke, however, the rest of the results were showing tendency to benefits however they were not statistically significant. The composite primary outcome HRs  is 0.91 (95% CI 0.78–1.06; p = 0.21) for cardiovascular death, 0.96 (0.79–1.16; p = 0.65) for non-fatal myocardial infarction, and 0.76 (0.61–0.95; p = 0.017) for non-fatal stroke. In the sub analysis obese patients (BMI > 32) benefited more than those with BMI < 32. [57].

On the other hand, SGLT2i have a robust and consistent effect on the prevention of HF and renal outcomes than on the atherosclerotic CV events. However, treatment with SGLT2i appears to result in a moderate reduction in the risk of MACE, no effect has been observed in patients with multiple risk factors for ASCVD [25–28]. In the DECLARE-TIMI 58 trial [28], the cohort included 60% patients with no previous CVD. It is very important to note that the statistically significant primary outcome benefits in this trial were mainly derived by reduction in the HHF, while cardiac death was similar between the dapagliflozin and placebo group (HR: 0.98; 95% CI 0.82–1.17; p = 0.005). The dapagliflozin also reduced composite renal outcomes significantly by 24% (HR: 0.76; 95% CI 0.67–0.87; p-value not reported).

Few studies on SGLT2i have reported a possible increased risk of stroke, amputation, and fractures [26, 27, 55, 56]; however, the DECLARE-TIMI 58 trial [28] did not show any such evidence. Moreover, despite an excess cases of bladder cancer in the earlier, smaller dapagliflozin studies, the observed rate of bladder cancer in DECLARE-TIMI 58 was lower with dapagliflozin than with placebo [28].

The CVD-REAL trial (Comparative Effectiveness of Cardiovascular Outcomes in New Users of SGLT2i) analysed a large contemporary real-world data regarding SGLT2i that was obtained from the clinicians across several countries [57]. In this trial, the SGLT2i were associated with a 39% relative risk reduction in HHF, a 46% reduction in the HHF or death composite, and a 51% reduction in all-cause death compared to other AHAs. Since approximately 87% patients did not have known CVD at the randomization, the lower rates of HHF and death associated with the SGLT2i treatment are likely class related, suggesting that the benefits of SGLT2i on the prevention of HF may extend to lower-risk patients than those enrolled in randomized trials so far.

It is advisable to initiate the GLP1 analogue (dulaglutide) in obese type 2 without CHF as a second line therapy if the HbA1c target is not achieved by the first line. However, in case of high risk for CVD, or renal issues, one can choose the best out of the SGLT2i and GLP1-RA. If the first category doesn’t effectively work in these patients, switching over to the second category is recommended as a third line treatment.

#### Recommendation: we recommend using pioglitazone (thiazolidinedione) as a fourth line therapy in patients with T2DM and high risk for CVD (Grade A)

In patients with T2DM, pioglitazone has shown benefits in reducing HbA1c, improving insulin sensitivity, and reducing CV events; compared to other medications, the benefits were sustained over a longer period of time [32]. The combination therapy of pioglitazone with exenatide or metformin, studied by Abdul-Ghani et al. [58, 59], was associated with persistent and significant lowering of HbA1c at 6 months to 2 years. This glycaemic benefit was achieved on top of a 7.5-fold lower rate of hypoglycaemic events when compared to conventional therapy. Interestingly, in the ACT NOW trial (Actos Now for Prevention of Diabetes), pioglitazone showed delayed progression to T2DM by 72% in patients with impaired glucose tolerance [60].

In the PROactive trial [32], incidences of peripheral revascularizations and amputations occurred slightly more often in the pioglitazone group. However, in patients who previously had a stroke, pioglitazone significantly reduced the risk of fatal or non-fatal stroke (HR: 0.53, 95% CI 0.34–0.85; p = 0.0085). Based on these results, another CVOT (IRIS trial) was conducted, that consisted patients with insulin resistance (majority pre-diabetics) and demonstrated a highly significant reduction in primary endpoint (fatal and non-fatal MI or fatal and non-fatal stroke), but there was no difference in terms of total stroke incidences, HHF, and all-cause mortality [34].

With all the data collected and analysed, we recommend using pioglitazone with a cautious weighing of the negative effects and considered as a fourth line therapy in patients at high risk for CVD; those with insulin resistance, previous stroke or pre-diabetes; and in patients with T2DM with or without ASCVD.

#### Recommendation: if high-risk patients did not achieve patient specific HbA1c target, we recommend using either DPP4 inhibitors (DPP4i), sulphonylureas, glinides, acarbose (α-glucosidase inhibitor), or insulin (Grade B, C)

Overall, the DPP4i have been observed to be neutral regarding CVD risk as per the 3-point MACE composite outcomes [22–24]. However, saxagliptin and alogliptin were associated with a higher risk of HHF (see Table 2), and their package inserts now have cautions about HF [61, 62]. There was also a trend seen towards higher all-cause mortality with saxagliptin and in the subsets of patients receiving alogliptin [22, 24]. Therefore, until we have generous data regarding their safety, sitagliptin and linagliptin seem to be the agents of choice in patients with T2DM and high risk of CVD on the basis of their results.

It has been widely believed that insulin therapy may be associated with atherosclerosis and an increased risk of CVD. However, according to the CVOTs (see Table 2), insulin glargine and degludec appeared to be neutral in terms of CV risks [33, 35]. Insulin degludec was also non-inferior to insulin glargine for the primary outcome of CVD, MI, or stroke. It is however noteworthy that the primary adverse effect observed with insulin glargine was hypoglycaemia (three times more than placebo) and the incidence of severe hypoglycaemia may increase the risk of death for up to 1 year after its occurrence, as mentioned in the CVOTs. Another point to be noted is that in the DEVOTE trial [35], 40% patients were not on metformin at baseline. Given that such a large number of trial participants had no exposure to metformin at all, the results of such CVOTs should not be interpreted exclusively as adding the novel therapy to metformin, but instead as effects on CV outcomes independent of metformin use.

The ACE trial for acarbose versus placebo showed neutral results in reducing CV death, non-fatal MI, non-fatal stroke, or hospitalisation for unstable angina or HHF [36]. Despite this, there is not enough data available regarding this class of AHAs stating CV safety.

Though glinides have been studied in the pre-diabetic population and observed to significantly reduce the risk of developing new-onset T2DM or CV complications [63], no studies have been carried out to demonstrate their effect in T2DM patients with associated high CV risk or with established HF/ASCVD + HF.

As of now, there are no available trials on CV safety of sulphonylureas. A recent observational analysis supports the current concerns regarding potential adverse effects of sulfonylureas on CV outcomes [64].

### Management of T2DM in patients with ASCVD

**Fig. 2:**
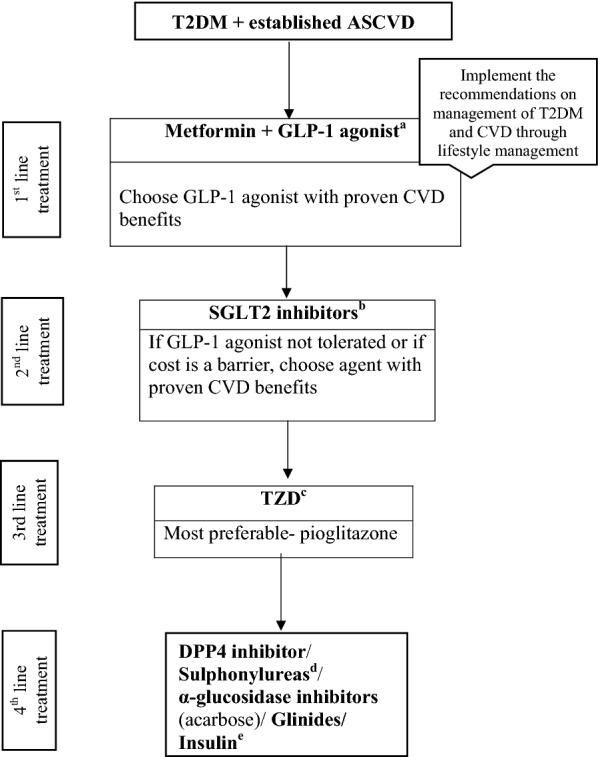
Evidence-based algorithm for the management of patients with T2DM and established ASCVD. *ASCVD* atherosclerotic cardiovascular disease, *GLP-1* glucagon-like peptide-1, *SGLT2* sodium–glucose transporter proteins-2, *TZD* thiazolidinedione, *DPP4* dipeptidylpeptidase-4. ^a^Proven CVD benefits means the agent has a label indication of reducing the CVD events. For GLP-1 agonist evidence based preference is Liraglutide > Semaglutide > Exenatide > Lixenatide. Caution to be exercised in case of end-stage renal disease. ^b^For SGLT2 inhibitors evidence based preference is Empagliflozin > Canagliflozin. ^c^Low dose TZDs are better tolerated. To be cautiously added to the patients with no history of heart failure and active surveillance to be maintained throughout the treatment. ^d^Choose later generation SU to minimize the risk of hypoglycaemia. ^e^Degludec and insulin Glargine (U100) have shown CVD safety

In patients with established ASCVD, there is a good evidence that the use of GLP1-RA (semaglutide and liraglutide), SGLT2i (empagliflozin and canagliflozin), and thiazolidinedione (pioglitazone) are associated with beneficial reduction in CV risk. However, pioglitazone should be used cautiously in patients with or at very high risk for HF [15, 16, 26, 27, 32]. Figure 2 demonstrates the treatment algorithm with the AHAs in this set of population for patient-specific glycaemic control.

#### Recommendation: we recommend using GLP1-RA along with life style changes and metformin as a first line therapy (Grade A)

The LEADER, SUSTAIN-6, and REWIND are the positive trials in the GLP1-RA class [15, 16, 54, 65]. The LEADER trial showed a significant reduction in stroke, CV mortality, and all-cause mortality; and a non-significant reduction in non-fatal stroke, MI, and HHF [16]. Similarly, the SUSTAIN-6 trial showed a significant reduction in primary composite endpoint, and non-fatal stroke; while the non-fatal MI and CV death were the same between the semaglutide and placebo group [15]. In the EXSCEL trial [31], exenatide was found to be non-inferior to the placebo, the primary composite endpoint was not reaching superiority. All-cause death was lower in the exenatide but not statistically significant. In the ELIXA trial, Lixisenatide did not demonstrate CVD benefit or harm in patient recruited 6 months following the acute MI [30].

The Kaplan–Meier curves in GLP1-RA trials showed CV benefit within 12–18 months (indicating anti-atherogenic benefits) [15, 16, 30, 31]. Postulations for the underlying mechanism of the anti-atherogenic effect of GLP1 analogues included the anti-inflammatory action. Some studies have shown that in non-obese T2DM patients during euglycaemic clamp or euinsulinaemic clamp, infusion of GLP1 analogues reduced the concentration of interleukin-6, prostaglandin F2α, intracellular adhesion molecule-1, and nitro-tyrosine [66]. Other proposed mechanisms include increased cardiac muscle glucose uptake, increased heart rate and hence enhanced left ventricular function. A similar effect is seen on blood vessels, under the influence of GLP1 analogues there will be an attenuated inflammatory response, decreased platelet aggregation, better vasodilatation, increased blood flow, and less smooth muscle proliferation with resultant plaque stability [67]. Lastly, the beneficial effect of GLP1 analogues is mediated through reduction in blood pressure and improvement lipid profile [68, 69].

#### Recommendation: we recommend using SGLT2i as second line therapy in patients with ASCVD (Grade A)

If GLP1-RAs are not tolerated or cost is a barrier to the treatment, SGLT2i would be a viable alternative. In the CVD-REAL study, the use of SGLT2i, against the oral glucose lowering drugs, was associated with lower rates of HHF (HR: 0.61; 95% CI 0.51–0.73; p < 0.001) and all-cause mortality (HR: 0.49; 95% CI 0.41–0.57; p < 0.001). These results are remarkably similar in real-world practice to those seen in the EMPA-REG OUTCOME trial [26, 56]. Furthermore, there was no significant heterogeneity in results across countries despite geographic variations in the use of specific SGLT2i, suggesting that the associated lower risks for CV outcomes were likely class related.

The CVOT trial of EMPA-REG [26] and CANVAS [27] demonstrated comparative results with empagliflozin and canagliflozin. Both the agents had reduced the 3-point MACE by 14%, and HHF by 35% (empagliflozin) and 33% (canagliflozin). Empagliflozin was associated with a signification reduction in CV death and all-cause mortality (38% and 32%), unlike canagliflozin (13% and 10%). Furthermore, the CV benefits of canagliflozin were diluted by the adverse events noticed in the CANVAS trial, mainly the increased risk of amputation, diabetic ketoacidosis, and increased risk of fractures (see Table 1).

The Kaplan–Meier curves in case of SGLT2i CVOT trials indicated benefits from the intervention as early as 12 weeks [26–28]; this clearly indicates that the benefits of the SGLT2i in achieving CV outcomes in patients with T2DM (all-cause mortality and HF) were not related to improved glycaemia. The exact mechanism of action is unknown; however, many postulations have been suggested including the haemodynamic and natriuretic effect which results in reduced ventricular load [70, 71]. Some authors attributed the benefits to the switch in myocardial substrate (super fuel theory) [72, 73], while other considered the modulation of adipokine production [74, 75] and cardiac remodelling [76].

Apart from the CV benefits, the SGLT2i also showed satisfactory renal outcomes in primary and secondary settings [77, 78]. Considering all these advantages, American Diabetes Association and the European Association for the Study of Diabetes have regarded SGLT2i as second-line therapy in patients with T2DM and ASCVD [47].

#### Recommendation: we recommend using pioglitazone (thiazolidinedione) as a third line therapy in patients with ASCVD, and no element of HF (Grade A)

In addition to the benefits discussed earlier (see the recommendation on pioglitazone as a third line therapy in T2DM patients with high risk of CVD), it is also observed that overall pioglitazone lowers the risk of important atherosclerotic events (see Table 2). However, the incidences of congestive heart failure, which was an exclusion criterion in the PROactive trial [32], were substantially higher in patients who received pioglitazone compared to placebo. Furthermore, the IRIS trial [34] confirmed that weight gain can be substantial with pioglitazone (weight gain > 10 lb in 50% patients and > 30 lb in 11.4% patients on pioglitazone). Additionally, there were 3.7% more pioglitazone-related bone fractures along with more frequent peripheral oedema compared to placebo.

Considering all the pros and cons, we therefore suggest doing an active surveillance of patients against the risks of HF, peripheral oedema, weight gain, and fractures from falls before initiating pioglitazone.

#### Recommendation: we recommend using one of the following options if the glycaemic targets are yet not met: DPP4i, sulphonylureas, acarbose (α-glucosidase inhibitor), glinides, or insulin (Grade B, C)

In patients with ASCVD, DPP4i do not seem to have any harmful effect on major adverse CV outcomes and risk for HF, except for saxagliptin and alogliptin, which were associated with an increased risk for HHF, predominantly in patients with CKD, pre-existing HF, or elevated levels of natriuretic peptides (NPs) at baseline. Though these agents have demonstrated positive CV effect, there is no enough data that supports CV risk reduction in patients with clinical ASCVD [22–24].

Given the lack of regulatory requirement to prove CV safety of insulins, there is little to no incentive to study the CV safety and efficacy of short-acting insulins in dedicated CVOTs, and their role in the management of patients with T2DM and prevalent ASCVD remains uncertain [33, 35].

Other AHAs mentioned in the recommendation have been discussed already in the previous section of recommendations (DPP4i, sulphonylureas, glinides, acarbose, or insulin for high-risk patients not achieving the HbA1c target).

### Management of T2DM in patients with HF or ASCVD and HF

**Fig. 3:**
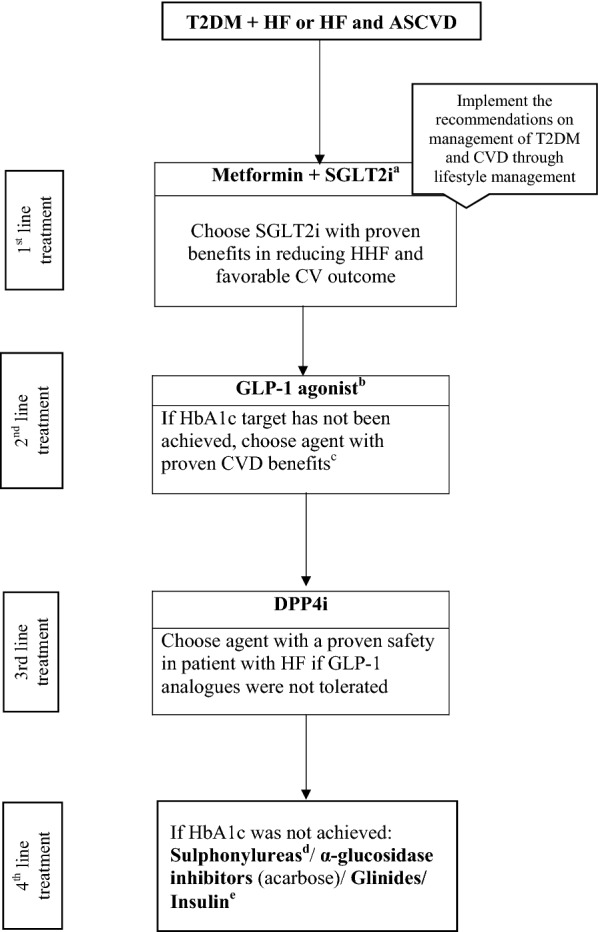
Evidence-based algorithm for the management of patients with T2DM and HF or HF + ASCVD. *ASCVD* atherosclerotic cardiovascular disease, *HF* heart failure, *GLP-1* glucagon-like peptide-1, *SGLT2* sodium–glucose transporter proteins-2, *DPP4* dipeptidylpeptidase-4. ^a^both empagliflozin and canagliflozin have shown reduction in HF in CVOT trials. ^b^For GLP-1 agonist evidence based preference is Liraglutide > Semaglutide > Exenatide > Lixenatide. Caution to be exercised in case of end-stage renal disease. ^c^Proven CVD benefits means the agent has a label indication of reducing the CVD events. ^d^Choose later generation SU to minimize the risk of hypoglycaemia. ^e^Degludec and insulin Glargine (U100) have shown CVD safety. Avoid thiazolidinediones, saxagliptin or Alogliptin in patients with ASCVD and HF

The treatment algorithm with AHAs for T2DM patients with HF or HF + ASCVD is depicted in Fig. 3.

#### Recommendation: we recommend assessing the eGFR and myocardial systolic function in patients with ASCVD early in the treatment paradigm (Grade A)

Estimation of the eGFR is essential as the data from different trials have shown that benefits in HF are dependent on the eGFR [79, 80]. It was also seen in the initial trials of SGLT2i that when the eGFR drops below 45 mL/min/1.73 m^2^, the beneficial effect on HbA1c would start to fade; however, a growing body of evidence suggests the persistence of CV and renal benefits beyond that level [81].

Many ongoing studies are evaluating the effect of SGLT2i on HF. Some are assessing it on the HF with preserved ejection fraction (HFpEF) while others on HF with reduced ejection fraction (HFrEF). When liraglutide was tested for left ventricular function in stable chronic HF patients with and without T2DM (LIVE trial) [82], it was found to be associated with serious adverse cardiac events and higher HF rates (HR: 3.9; 95% CI 1.1–13.8; p = 0.029). The FIGHT trial (Functional Impact of GLP-1 for Heart Failure trial) evaluated effect of liraglutide in patients with systolic HFrEF who were recently hospitalized for HF. The results demonstrated no association between liraglutide and increased cardiac events [83]. Interestingly, data from LEADER trial [16] showed a non-significant reduction in HF. This, in turn, explained that the difference in ejection fraction plays a vital role in predicting the CV outcomes.

#### Recommendation: in patients with HF and ASCVD, we recommend starting an SGLT2i with proven benefits and metformin as a first line therapy to reduce HHF along with life style changes (Grade A)

In patients with history of ASCVD and HF, evidences support the use of SGLT2i along with metformin and life style changes [26, 27]. In the EMPA-REG OUTCOME trial [26], empagliflozin was associated with highly significant 35% relative risk reduction in HF in patients with previous ASCVD. Also, in the first 6 months of empagliflozin treatment, there was a significant improvement in the LV function of the T2DM patients as observed in the EMPA-HEART study [84]. Similarly, canagliflozin in CANVAS trial reduced the relative risk of HHF by a highly significant 33%.

Interestingly, in case of EMPA-REG and CANVAS trials [26, 27], the Kaplan–Meier curves demonstrated a significant lower risk of CV death and even lower risk for HF incidences. Hence, empagliflozin or canagliflozin can be effectively used as a first line therapy.

#### Recommendation: if patient-specific HbA1c target has not been achieved we recommend using GLP1-RA as a second line therapy (Grade A)

We recommend using GLP1-RA as a second line therapy in patients with ASCVD and HFpEF. This recommendation was based on the results from LEADER trial [16] that showed a non-significant 14% reduction in HHF. On the other hand, data from SUSTAIN 6 trial [15] has shown a non-significant 11% increase in the risk of HF. It is therefore essential to ensure the type of HF and the ejection fraction before starting the GLP1-RA.

#### Recommendation: we recommend using a DPP4i with a safety record in regards to HF as a third line therapy if GLP1 analogues were not tolerated (Grade A)

If after 3 months the patient-specific target HbA1c was not achieved, a consideration of DPP4i with known CV safety is recommended, provided the patient is not already on a GLP1 analogue. The CVOTs of three DPP4i-saxagliptin (SAVOR-TIMI 53) [22], alogliptin (EXAMINE) [24], and sitagliptin (TECOS) [25] showed that these agents were non-inferior to the placebo regarding the primary CV end point; however, none of them demonstrated superiority. The only difference between the three trials is that the EXAMINE and SAVOR-TIMI 53 trials evaluated primary composite outcomes of CV death, non-fatal MI, or non-fatal stroke; while the TECOS trial used an additional endpoint of hospitalisation for unstable angina in the primary composite outcome. These trials showed heterogeneous effect on HHF. In the SAVOR-TIMI 53 trial [22], saxagliptin was associated with a significant 27% higher risk of HHF compared with placebo, alogliptin showed a 19% non-significant increase in the relative risk of HF in the EXAMINE trial [24], whereas the results from TECOS trial [25] indicated sitagliptin to be CV neutral.

#### Recommendation: if patient-specific HbA1c was not achieved we recommend using sulphonylureas, glinides, acarbose (α-glucosidase inhibitor), or insulin (Grade B, C)

These agents are discussed in detail under the sections ‘management of T2DM in patients at a risk of CVD’ and ‘management of T2DM in patients with ASCVD’.

#### Recommendation: we recommend against using pioglitazone, saxagliptin, or alogliptin in patients with ASCVD and HF (Grade A)

In the PROactive trial [32], the main side effects of pioglitazone reported were weight gain, oedema, and bone fracture. Though the IRIS trial had lesser and comparable cases of HF in both pioglitazone and placebo groups, this observation was attributed to the exclusion of patients with a history of HF and the use of safety algorithms [34]. Meanwhile, data from SAVOR-TIMI [22] and EXAMINE trial [24] also showed an increased risk of HHF (see Table 2).

### Special circumstances

#### Recommendation: in patients with retinopathy, we recommend starting treatment with metformin or SGLT2i (Grade A, B)

The UKPDS trial [11] showed a significant 37% reduction in the microvascular complications with the use of metformin. It also significantly lowered the risk of diabetic retinopathy progression in overweight diabetic patients. After that, several studies were carried out focusing on influence of metformin on retinopathy in patients with high-risk/established T2DM [85–87] which demonstrated a significant association between long term metformin use (≥ 5 years) and a reduced risk/severity of diabetic retinopathy in these patients. This effect of metformin is assumed to be linked to the drug-induced restoration of microvascular energy balance through the activation of AMPK [85].

The EMPA-REG trial [26] demonstrated a significant relative risk reduction of 38% in the pre-specified composite microvascular outcomes. The incidence of retinopathy development was very less in this trial with 1.6% of patients on empagliflozin affected by it (incidence rates: 5.6/1000 patient-years) compared to placebo (2.1%). Having said that, it is important to note that this composite outcome was driven entirely by the renal component. Hence, extrapolation of these results would need validation in more diverse population [88].

The GLP1-RA were associated with an increased risk of retinopathy as shown in SUSTAIN 6 trial [15], in which the risk of retinopathy complications such as vitreous haemorrage, onset of diabetes-related blindness, and the need for treatment with an intra-vitreal agent/retinal photocoagulation had increased by 76% (HR: 1.76; CI 1.11–2.78; p = 0.02). Alternatively, liraglutide was associated with 15% increase in the risk of retinopathy; however, it was not statistically significant [16].

#### Recommendation: in patients with CKD having either an eGFR < 60 mL/min/1.73 m^2^ or albuminuria, we recommend adding SGLT2i to standard treatment of diabetic nephropathy. We further recommend using GLP1-RA as a second line following SGLT2i (Grade A, B)

The renal outcomes of the AHAs have been summarized in Additional file 1: Table S1. Patients with T2DM carry two to fourfolds’ risk of developing CV mortality, and once they develop proteinuria the risk increases to eightfolds compared to general population. Interestingly, it has been reported that patients with T2DM are 16–60 times more likely to die of premature heart disease than to reach end stage renal disease. Once eGFR declines below 60 mL/min/1.73 m^2^, the risk for death, major CV events, and hospitalisation increases [89].

Diabetic kidney disease occurs in a continuum that starts with the risk factors of hypertension, T2DM, smoking, and metabolic syndrome progressing to microalbuminuria, macroalbuminuria, mild/moderate/end stage renal disease, and dialysis followed by renal transplant [90]. Tight glycaemic and blood pressure control are very effective in reducing microvascular complications and diabetic nephropathy [10, 11]. SGLT2i are the novel interventions in management of diabetic nephropathy, data from the EMPA-REG outcomes trial [26] showed that empagliflozin resulted in 39% risk reduction of new onset or worsening nephropathy (HR 0.61; CI 0.53–0.70; p < 0.001) and CV death (HR: 0.61; CI 0.55–0.69; p < 0.001). More significant findings were 44% risk reduction in doubling of serum creatinine (HR: 0.56; CI 0.39–0.79; p < 0.001), 55% reduction in initiation of renal replacement therapy (HR: 0.45; CI 0.21–0.97; p < 0.001) or death from renal disease (HR: 0.61; CI 0.55–0.69; p < 0.001). Dapagliflozin has shown similar beneficial effects in the DECLARE-TIMI 58 trial [28]—it reduced the composite renal outcomes in high risk patients by 24% [14]. Data from the CANTATA-SU trial (CANagliflozin Treatment And Trial Analysis-Sulfonylurea) [91] showed that the use of canagliflozin was associated with initial decreases in eGFR, which then stabilized from week 12 to 52; whereas in the glimepiride arm, there was a progressive decline observed.

It is important to note that renal outcomes in the GLP1-RA trials were positive; however, they did not mount to the benefits demonstrated by SGLT2i (Additional file 1: Table S1). In the LEADER trial [16], the composite renal and retinal outcomes were reduced by 16% (HR: 0.84; 95% CI 0.73–0.97; p = 0.02), whereas the renal outcomes individually were reduced by 22% (HR: 0.78; 95% CI 0.67–0.92; p = 0.003). Similar results were seen in SUSTAIN 6 trial [15], semaglutide reduced the new onset or worsening nephropathy by 36% (HR: 0.64; CI 0.46–0.88; p = 0.005).

As mentioned above, following the approach of tight glycaemic control along with blood pressure regulation has a beneficial effect on management of renal complications. The clinical effects of the AHAs can be significantly achieved on top of the standard treatment with anti-hypertensive agents [26]. Blocking the renin–angiotensin–aldosterone system is currently considered the gold standard treatment for diabetic nephropathy. Studies have proved the efficacy of this class of agents in treating various stages of renal continuum. The TRENDY trial (Telmisartan versus Ramipril in renal ENdothelium DYsfunction) [92] has shown effectiveness of angiotensin-converting enzyme (ACE) inhibitors and angiotensin-II receptor blockers (ARBs) in improving endothelial dysfunction. Furthermore, many trials have shown the potential of ACE inhibitors and ARBs in preventing microalbuminuria as well as preventing the progression to macroalbuminuria [93–97]. In the IDNT trial (The Irbesartan Diabetic Nephropathy Trial) [98], irbesartan reduced the progression to end stage renal disease by 23%, while in RENAAL trial (Reduction of Endpoints in NIDDM with the Angiotensin II Antagonist Losartan) [99], losartan resulted in 29% risk reduction in progression to end stage renal disease and delay in doubling of serum creatinine.

#### Recommendation: in T2DM patients with peripheral vascular disease, we suggest the use of GLP1 analogues and to avoid canagliflozin (Grade A, B)

In T2DM patients with CVD and peripheral vascular disease, we suggest the use of GLP1-RA as the data from LEADER and SUSTAIN-6 did not show any evidence of increased risk of amputation, unlike canagliflozin, which showed an almost double the risk of lower limb amputation [15–17]. Fortunately, this risk has not been proved in any other studies of the SGLT2i including the DECLARE- TIMI 58 trial [28].

## Consensus recommendations for management of CVD in patients with T2DM

Although CVD is an umbrella term, this section of the document mainly summarizes the prevalence and pathophysiology of the underlying intersection between T2DM and HF; along with the contemporary treatment options.

A population-based study conducted by Shah et al. [100] in patients with T2DM without overt CVD demonstrated that incident HF was observed more frequently (14.1%) than the vascular events, including MI or stroke. The T2DM is hence recognised as an independent risk factor for the development of HF. In the Kaiser Permanente study [101], patients with T2DM aged < 75 years had an approximately threefold higher prevalence of HF than those without T2DM. The T2DM patients between 75 and 84 years of age were associated with doubling of risk for HF.

The clinical symptoms of HF are characterised as very typical (breathlessness, ankle swelling, and fatigue) that could be accompanied by signs (elevated jugular venous pressure, pulmonary crackles, and peripheral oedema) due to a structural and/or functional cardiac abnormality (left ventricular systolic dysfunction [HFrEF] or diastolic dysfunction [HFpEF]), which in turn fails to deliver oxygen to the metabolising tissues [102, 103].

Based on the measurement of left ventricular ejection fraction (LVEF) function, the HF patients are divided into two groups: (a) those with normal LVEF (≥ 50%; in case of HFpEF) and (b) those with reduced LVEF (< 40%; in case of HFrEF). The diagnosis of HFpEF is observed to be more challenging than the diagnosis of HFrEF. Patients with HFpEF generally do not have a dilated LV, but instead often have increased LV wall thickness and/or left atrial size as a sign of increased filling pressures. Most of the patients additionally have impaired LV filling or suction capacity, categorised as diastolic dysfunction, which is generally accepted as the likely cause of HF in these patients [102, 104].

As observed in a study reported elsewhere, a significant proportion of patients with HFrEF and HFpEF have unrecognised diabetes mellitus, and in patients diagnosed with HF, the pre-diabetes or diabetes mellitus is often missed [105, 106]. It is therefore important to screen patients with HF for undiagnosed glucose intolerance or T2DM since the novel therapies offer opportunities to improve the clinical outcomes. Table 2 depicts the AHAs that significantly reduce the CV mortality and HHF in T2DM patients.

### Pathophysiological aspects of T2DM and HF [107]

In patients with T2DM, the most common CV risk factors that causes HF are CAD and hypertension. It is also stated that the physiological damage caused by T2DM can lead to HF by directly affecting the structure and function of the heart. The insulin resistance or hyperinsulinaemia, impaired glucose tolerance or hyperglycaemia, and their consequent maladaptive responses result in myocardial dysfunction in the people even years before overt T2DM develops [108]. These major drivers result in increased free fatty acid release, cardiomyocyte contractile dysfunction, mitochondrial network fragmentation, and an increase in protein kinase-C activity; thereby causing myocyte alterations [109–112]. They further lead to the activation of reactive oxygen species and the deposition of advanced glycosylation end products in both endothelial and smooth muscle cells, which predisposes to concentric LV remodelling and raises the LV diastolic stiffness [110, 111]. Moreover, the degree of glucose dysregulation correlates with the severity of LV diastolic dysfunction [113], and increased risk of incident HF and CV mortality in T2DM patients [114–116]. Almost half of HF patients with T2DM have HFpEF, which is difficult to diagnose because the symptoms are often mild, appear only upon physical activity, and could be misdiagnosed as chronic obstructive pulmonary disease [117].

#### HFpEF and HFrEF pathophysiology in T2DM

HFpEF is usually associated with mild/early stage T2DM complications, whereas HFrEF is associated with more severe complications of T2DM. This implies that the severity and duration of hyperglycaemia are important for the development of LV dysfunction [118, 119].

As generally observed, the pathophysiology for the development of HFrEF consists of cardiomyocyte loss caused by ischaemia or toxic agents [118, 120, 121]. On the other hand, the underlying pathophysiology of HFpEF is diverse, which is associated with different phenotypes including various concomitant CVDs (e.g. atrial fibrillation, arterial hypertension, CAD, pulmonary hypertension) and non-CVDs (diabetes, CKD, anaemia, iron deficiency, chronic obstructive pulmonary disorder, and obesity). Compared to HFrEF, the HFpEF condition leads to hospitalisation and death in the patients due to non-CV reasons [120, 121].

### Screening and assessment of HF in patients with T2DM [104, 122–124]

**Fig. 4:**
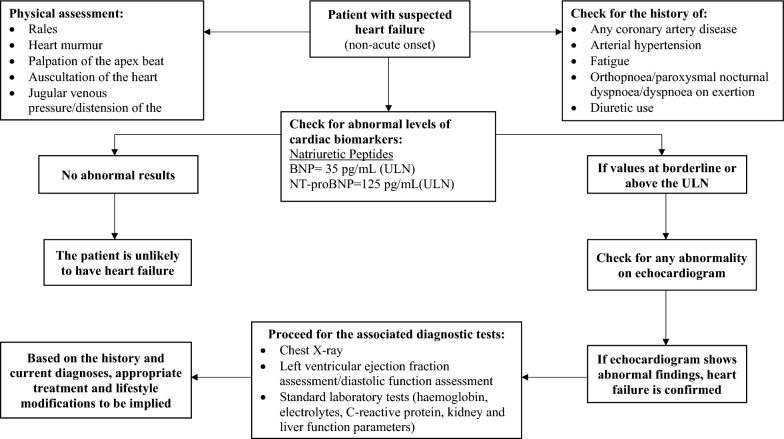
Screening approach for the T2DM patients with suspected heart failure. *BNP* brain natriuretic peptide, *ECG* electrocardiogram, *NT-proBNP* N-terminal pro-brain natriuretic peptide, *ULN* upper limit of normal

As mentioned earlier, diagnosing HF is complex due to its significantly diverse aetiology and clinical heterogeneity. A general approach followed globally for the screening of HF is depicted in Fig. 4. The diagnostic pathways require observation of typical signs and symptoms along with raised HF-related biomarkers such as brain natriuretic peptide (BNP) or N-terminal pro-brain natriuretic peptide (NT-proBNP) and presence of abnormal imaging findings to determine the cause and assess the severity of myocardial dysfunction. Importantly, biomarkers are not specific for HF—there is a vast number of differential diagnoses such as advanced age, non-HF related cardiac causes (acute coronary syndromes, myocarditis, etc.), diabetes, respiratory disease, renal or hepatic dysfunction, and many other that may cause their elevation. Therefore, while raised NPs are helpful diagnostically and relevant prognostically, a detailed evaluation to ensure their diagnostic accuracy in HF is mandatory.

#### Cardiac biomarkers and imaging techniques

NPs are secreted into the circulation as a result of increased volume expansion and/or pressure and stiffness in the cardiac muscles. Regardless of the underlying aetiology, they are comparable in patients with HFrEF and HFpEF [125]. In an analysis of over 1000 patients with T2DM and concomitant HF, interestingly, the levels of NT-proBNP and troponin were generally higher in patients with T2DM [122]. Co-morbidities such as earlier (premature) diagnosis of HF, ischaemia, hypertension, and kidney disease that are prevalent in the T2DM lead to altered levels of NPs and hence, these confounding factors need to be taken into account when interpreting NP results in T2DM patients.

Patients with normal plasma NP levels are unlikely to have HF. The upper limit of normal for BNP and NT-proBNP is 35 pg/mL and 125 pg/mL, respectively, in the non-acute onset setting; while, in the acute setting, it is generally higher [BNP: 100 pg/mL, NT-proBNP: 300 pg/mL]. Diagnostic values apply similarly to HFrEF and HFpEF; on an average, values are lower for HFpEF than for HFrEF [126, 127].

Due to better accuracy and proven superiority, the BNP/NT-proBNP and troponin biomarkers are widely used in comparison to other cardiac biomarkers (copeptin, CT-proET-1, hs-CRP, procalcitonin, PAI-1, galectin-3, cystatin-C). However, there is an active ongoing research in this field and future targets such as micro-RNAs seem promising in improving the diagnostic accuracy [128].

Since NPs are highly sensitive but lack specificity, values beyond the normal range require further investigation for their clinical relevance. As a result, imaging techniques are used to complement NPs and provide additional clarity in the phenotypic evaluation.

Cardiac imaging plays a vital role in diagnosing HF and further treatment guidance. Of several imaging techniques available, echocardiography is preferred in patients with suspected HF due to its accuracy, availability/portability, safety, and cost. It can accurately assess and very well differentiate between the HF, diastolic dysfunctions, valvular heart diseases, and coronary heart disease [123, 129, 130]. For an instance, since the guidelines did not stress on its screening, the prevalence of undiagnosed coronary heart disease is observed to be considerable (23–31%), which can eventually lead to HF development. In this case, the echocardiography can sensitively detect the difference between an established HF and a coronary heart disease that is gradually leading to HF [102, 103].

Patients with a completely normal electrocardiogram (ECG) are unlikely to have HF. Having said that, an abnormal ECG cannot confirm the diagnosis of HF due to low specificity, it only increases likelihood of its diagnosis [131–134]. Therefore, the routine use of an ECG is mainly recommended to rule out HF.

Two-dimensional transthoracic echocardiography and tissue Doppler imaging can detect the presence of LV hypertrophy and also provide a significant differentiation between HFpEF and HFrEF. However, suboptimal image quality, body habitus, high inter- and intra-observer variability, and limited spatial resolution limit its use [135].

Cardiac magnetic resonance imaging (CMR imaging), being an advanced modality, offers an improved functional and morphological assessment such as tissue characterisation and assessment of cardiac tissue perfusion, oedema, energy metabolism, and fibrosis [136]. CMR imaging thus facilitates symptom correlation with underlying disease-specific pathophysiology which can strengthen our understanding of the HF aetiology and the potential impact of concurrent T2DM [137, 138].

### Treatment approaches for HF in patients with T2DM: a brief overview [102, 104, 139–141]

Multiple recent trials with SGLT2i have found significant reduction in HHF [26–28]. Such clinical benefits of the AHAs are frequently seen in these CVOTs (Table 2) that have been attributed to various mechanisms such as urinary volume loss along with sodium and sugar loss. A detailed discussion on preferable AHAs in patients with HF is previously mentioned in this document. There are no randomised clinical trials conducted to test the effect of CV interventions (drugs and/or devices) in T2DM patients with HF. However, enough evidences suggest that all interventions effective at improving prognosis in patients with HF are equally beneficial in patients with and without T2DM.

#### ACE-inhibitors and ARBs

The ACE-inhibitors in patients with HFrEF and T2DM have been shown to improve symptoms, and reduce hospitalisation and mortality. The ATLAS trial on lisinopril [142] in HFrEF patients with T2DM demonstrated a positive outcome for the composite primary endpoint (HHF or all-cause mortality).

The randomised clinical trials conducted on ARBs showed significant reduction in CV death, HHF, and all-cause mortality [143, 144]. However, a little is known about their tolerability in T2DM patients. It is further observed that ACE-inhibitors and ARBs may interfere with the renal potassium excretion; therefore, serum electrolytes monitoring including creatinine is recommended when starting or escalating the doses of these two drug classes.

#### Beta blockers

The beta-blockers in patients with T2DM and HF, in large randomised clinical trials, demonstrated significant improvements in morbidity and mortality that were comparable in patients without T2DM. Furthermore, a meta-analysis of several beta-blocker trials demonstrated to reduce all-cause mortality in patients with T2DM (HR 0.84) [145]. Also, the treatment benefits of beta-blockers in T2DM patients far outweigh the theoretical risks related to hypoglycaemia, slight changes in HbA1c along with serum lipids. These benefits, therefore, strongly support beta-blocker treatment in patients with concurrent T2DM and HF.

#### Mineralocorticoid receptor antagonists

Mineralocorticoid receptor antagonists are equally effective in patients with HF and T2DM; however, caution is necessary when these medications are used in patients with impaired renal function. Due to the frequent coexistence of diabetic nephropathy, a close surveillance of electrolyte as well as renal function is recommended to exclude the hyperkalaemia.

#### Sacubitril/valsartan

The sacubitril/valsartan combination was observed to be superior to the ACE-inhibitor enalapril in reducing the risks of death and HHF (primary endpoint) in patients with HFrEF as mentioned in the PARADIGM-HF trial [146]. It was also associated with a substantial HbA1c reduction and a lower rate of initiation of AHAs for T2DM compared to enalapril.

#### Nitrates and hydralazine

In the A-HeFT trial [147] (that included 41% of T2DM patients), the treatment effect of isosorbide dinitrate and hydralazine hydrochloride on mortality was comparable in patients with and without T2DM (HR: 0.56 and 0.59, respectively).

The two most recent drugs introduced in HF treatment, LCZ69 and ivabradine, are also effective in patients with T2DM and HF, and should be implemented as proposed by the guidelines of the European Society of Cardiology/Heart Failure Association [105].

As of now, there are no clinical trials examining the efficacy of diuretics in patients with both T2DM and HF. Therefore, their careful use is advised for symptomatic treatment to reduce ventricular filling pressures and correct pulmonary congestion.

## Discussion

Treatment of T2DM has now been expanded from a glucose-centric concept to an event-driven strategy due to the comorbidities. Many AHAs demonstrated effective CV and renal protection. The CVOTs, along with the management of glycaemia, have demonstrated to mitigate the microvascular and macrovascular risk. The SGLT2i and GLP1-RA show great promise in transforming the treatment of diabetes and concomitant CVDs by independently improving CV outcomes, over and above what can be achieved with standard of care management. Moreover, on the basis of researches conducted on the population with concomitant T2DM and CVDs, healthcare professionals have come up with a more reliable and novel screening techniques and therapeutic approaches for the CVDs as discussed in the earlier section. While these drugs seem promising in comorbidities management, their utility and safety in the general population remains an important parameter to be closely followed.

## Conclusions and future directions

Diabetes is currently considered a cardiovascular disease, given the fact that all complications are vascular (micro and macrovascular complications). Hence, management of diabetes and especially those with a history of CVD in a joint clinic by cardiologist and an endocrinologist would seem a logical approach. The authors, therefore, recommend a new T2DM/CVD clinic or a cardiometabolic clinic that follows a novel algorithm for the management of T2DM in patients with CVDs. The facilities provided at the clinic would be for T2DM patients with a history of HF or ischaemic heart disease, those who underwent percutaneous transluminal coronary angioplasty or Coronary artery bypass graft, and T2DM patients with abnormal BNP levels. The healthcare professionals with their expertise would look into managing HbA1c, low-density lipoprotein cholesterol target, blood pressure, body mass index, and elevated BNP levels to oversee the disease burden. Individual specific treatment algorithm designing and patient specific lifestyle modifications with regular follow-ups would be the key factors.

## Supplementary information

**Additional file 1: Table S1.** Summary of renal outcomes of the anti-hyperglycaemic agents.

## Data Availability

Not applicable.

## Abbreviations

ACE: Acarbose Cardiovascular Evaluation Trial
AHAs: anti-hyperglycaemic agents
ACE-inhibitors: angiotensin-converting enzyme inhibitors
ARBs: angiotensin-II receptor blockers
BNP: brain natriuretic peptide
CAD: coronary artery disease
CANVAS: CANagliflozin cardioVascular Assessment Study
ASCVD: atherosclerotic cardiovascular disease
CARMELINA: Cardiovascular and Renal Microvascular Outcome Study with Linagliptin in Patients with Type 2 Diabetes Mellitus
CKD: chronic kidney disease
CMR: cardiac magnetic resonance
CI: confidence interval
CVDs: cardiovascular diseases
CVOTs: cardiovascular outcomes trials
DECLARE-TIMI 58: Multicenter Trial to Evaluate the Effect of Dapagliflozin on the Incidence of Cardiovascular Events
DEVOTE: Cardiovascular Safety of Insulin Degludec Versus Insulin Glargine in Subjects with Type 2 Diabetes at High Risk of Cardiovascular Events
DPP-4i: DPP-4 dipeptidyl peptidase 4 inhibitors
ECG: electrocardiogram
eGFR: estimated GFR
ELIXA: Evaluation of Cardiovascular Outcomes in Patients with Type 2 Diabetes Mellitus after Acute Coronary Syndrome During Treatment with Lixisenatide
EMPA-REG: (Empagliflozin) Cardiovascular Outcome Event Trial in Type 2 Diabetes Mellitus Patients
EXAMINE: EXamination of cArdiovascular outcoMes with alogliptIN versus standard of care
EXSCEL: Exenatide Study of Cardiovascular Event Lowering Trial
GI: glycaemic index
GL: glycaemic load
GLP1-RA: glucagon like peptide1-receptor agonist
HARMONY: trial to evaluate effect of albiglutide on major adverse CV events in patients with T2DM and established CV disease
HHF: hospitalization for heart failure
HF: heart failure
HFpEF: HF with preserved ejection fraction
HFrEF: HF with reduced ejection fraction
HR: hazard ratio
IRIS: Insulin Resistance Intervention After Stroke Trial
LEADER: Liraglutide Effect and Action in Diabetes: Evaluation of cardiovascular outcome Results
LVEF: left ventricular ejection fraction
MACE: major adverse cardiovascular events
MI: myocardial infarction
MNT: medical nutritional therapy
NPs: natriuretic peptides
NT-proBNP: N-terminal pro-brain natriuretic peptide
ORIGIN: Outcome Reduction With Initial Glargine Intervention
PROactive: PROspective pioglitAzone Clinical Trial In macroVascular Events
RECORD: rosiglitazone evaluated for cardiovascular outcomes in oral agent combination therapy for type 2 diabetes mellitus
SAVOR-TIMI5325: Saxagliptin Assessment of Vascular Outcomes Recorded in Patients with Diabetes Mellitus-Thrombolysis in Myocardial Infarction 53375
SGLT-2: sodium–glucose cotransporter-2
SUSTAIN-6: Trial to Evaluate Cardiovascular and Other Long-term Outcomes With Semaglutide in Subjects With Type 2 Diabetes
TECOS: Trial Evaluating Cardiovascular Outcomes With Sitagliptin
T2DM: type 2 diabetes mellitus
UACR: urinary albumin:creatinine ratio
UKPDS: United Kingdom Prospective Diabetes Study
