# Supplementary material for: Consensus recommendations for management of patients with type 2 diabetes mellitus and cardiovascular diseases
Source: Diabetol Metab Syndr. 2019 Sep 26;11:80. doi: 10.1186/s13098-019-0476-0 (PMC6761728; doi:10.1186/s13098-019-0476-0)
Supplement: Supplementary file 1 — Additional file 1: Table S1. Summary of renal outcomes of the anti-hyperglycaemic agents. [file 13098_2019_476_MOESM1_ESM.docx]

**Additional file 1**

**Cardiovascular Diabetology**

**CONSENSUS RECOMMENDATIONS FOR CLINICAL PRACTICE IN PATIENTS WITH TYPE 2 DIABETES MELLITUS AND CARDIOVASCULAR DISEASES**

Bashier et al.

**Additional table**

Table S1. Summary of renal outcomes of the anti-hyperglycaemic agents.

| **Trial/Drug** | **Change in eGFR from baseline in mL/min/1.73m^2@^** | **Change in UACR from baseline in mg/g^@^** | **New-onset persistent macroalbuminuria** | **Doubling of serum creatinine** | **Progression to ESRD requiring RRT** | **Worsening nephropathy*** | **Composite renal outcome** |
| --- | --- | --- | --- | --- | --- | --- | --- |
|  | **HR (95% CI)** | | | | | | |
| SAVOR-TIMI 5325/  Saxagliptin [1,2] | Not reported | -34.3  (CI not available) | 2.2% vs 2.8%^§^ | 1.1  (0.89– 1.36) | 0.90  (0.61–1.32) | 1.08  (0.88–1.32) | 1.17^a^  (0.93–1.20) |
| CARMELINA/  Linagliptin [3] | -1.74  (-1.86 to -0.11) | -0.18  (-0.35 to -0.02) | Not reported,  (Micro^§^: 7.8% vs. 7.9%) | Not reported | 1.4% vs. 1.5%^§^ | 0.98  (0.82-1.18)** | Not reported |
| EXAMINE/  Alogliptin [4] | No difference^b^ | Not reported | Not reported | Not reported | 0.9% vs. 0.8%^§^ | Not reported | Not reported |
| TECOS/  Sitagliptin [5,6] | -1.34  (-1.76 to -0.91) | -0.18  (-0.35 to -0.02) | Not reported | Not reported | Not reported | Not reported | Not reported |
| EMPA-REG OUTCOME/  Empagliflozin [7-9] | 1.48  (CI not available) | Range:  -15% to -49%^c^ | 0.62  (0.54–0.72) | 0.56  (0.39–0.79) | 0.45  (0.21–0.97) | 0.61  (0.53–0.70) | 0.54^d^  (0.40–0.75) |
| Integrated CANVAS programme (CANVAS, CANVAS-R)/  Canagliflozin [10,11] | 1.2  (1.0–1.4) | -18%  (-16 to -20) | 0.73  (0.67–0.79) | 0.50  (0.30 –0.84) | 0.77  (0.30–1.97) | 0.60  (0.47–0.77)^#^ | 0.60^e^  (0.47–0.77) |
| DECLARE-TIMI 58/  Dapagliflozin [12] | -1.76  (-1.67 to −1.87) | Not reported | Not reported | Not reported | 0.53  (0.43−0.66) | 0.76  (0.67–0.87) | 0.69  (0.52–0.81) |
| Harmony/  Albiglutide | Data not available | | | | | | |
| LEADER/  Liraglutide [13,14] | Not reported | -17%  (-12 to -21) | 0.74  (0.60–0.91) | 0.89  (0.67–1.19) | 0.87  (0.61–1.24) | 0.78  (0.67–0.92) | 0.81^f^  (0.70–0.91) |
| SUSTAIN-6/  Semaglutide [15] | Not reported | Not reported | 0.54  (0.37–0.77) | 1.28  (0.64–2.58) | 0.91  (0.40–2.07) | 0.64  (0.46–0.88) | 0.79  (0.61–0.82) |
| ELIXA/  Lixisenatide [16] | No difference | –39.18%  (–68.53 to –9.84) | 0.81  (0.66 to 0.99) | 1.16  (0.74-1.83) | No difference | Not reported | 0.90  (0.70–1.05) |
| EXSCEL/  Exenatide [17] | Not reported | Not reported | 2.2% vs. 2.8%^§^ | Not reported | 0.7% vs. 0.9%^§^ | Not reported | Not reported |
| IRIS/  Pioglitazone [18] | -7 ±16  (-12.88 to -0.68)*** | 12 ±85  (-20.46 to 44.06)*** | Not reported | 0.05 ±0.14  (-0.001 to 0.10)^€^ | Not reported | Not reported | Not reported |
| PROactive/  Pioglitazone [19] | -0.8  (-0.8 to -0.4) | -24.8%  (-39.6 to -10.0) | Not reported | 1·67  (1·20–2·31) | 0.64  (0.53 to 0.75) | Not reported | Not reported |
| ORIGIN/  Insulin glargine [20] | Increased by 1.7 mL/min/1.73m^2^ in glargine group | -33.8%  (-24.6 to -43.8) | Not reported | Not reported | Not reported | Not reported | Not reported |
| DEVOTE/  Insulin degludec [21] | Not reported | Not reported | Not reported | Not reported | Not reported | 1.08  (0.88-1.32) | Not reported |
| ACE/  Acarbose [18] | 0 ±14  (-5.19 to 5.39)*** | -18 ±104  (-58.16 to 21.29)*** | Not reported | 0 ±0.13  (-0.06 to 0.04) ^€^ | Not reported | Not reported | Not reported |
| UKPDS/  Metformin [22] | Not reported | Not reported | Not reported,  Micro: 1.00 (0.77-1.30) | Not reported | 1.14  (0.09-14.94) | Not reported | 2.40  (0.22-26.39) |

eGFR: estimated glomerular filtration rate (mL/min/1.73m2); UACR: urinary albumin/creatinine ratio; ESRD: end-stage renal disease; RRT: renal replacement therapy; Micro: microalbuminuria; IQR: interquartile range; #Non-truncated integrated data (refers to pooled data from CANVAS, including before 20 November 2012 plus CANVAS-R). ^a^doubling of serum creatinine, dialysis, renal transplantation, serum creatinine>6mg/dL.

^b^basal eGFR (mL/min/1.73m^2^): ≥90, (-6.7 vs.-4.5); <90 but ≥60 (0.6 vs.1.0); <60 but ≥30, (1.1vs.2.1); < 30, (0.2 vs.1.6) ^c^Normo-albuminuria at baseline: (-15%, 95%CI: -22 to -7, P = 0.0004); microalbuminuria (micro) at baseline:

(-42%, 95%CI: -49 to -34, P < 0.0001), macroalbuminuria (macro) at baseline: (-49%, 95%CI: -60 to -36, P < 0.0001), ^d^Progression to macro: doubling of serum creatinine, initiation of renal replacement therapy (RRT), death due to renal disease; another post-hoc outcome was doubling of serum creatinine, initiation of RRT, death due to renal disease (HR:0.54,95%CI:0.40–0.75, P < 0.001), ^e^Sustained 40% reduction in eGFR, need for RRT, death due to renal causes; another composite end point was sustained doubling of serum creatinine, ESRD, death due to renal causes (HR:0.53,95%CI:0.33–0.84), ^f^Includes persistent doubling of serum creatinine, ESRD, death due to renal disease;

another composite endpoint was persistent doubling of serum creatinine, need for continuous RRT (ESRD; HR:0.85,95%CI:0.66–1.10, P = 0.20), ^@^Calculated as: HR (95% CI) of active drug minus HR (95% CI) of placebo, ^§^ % change from the baseline values has been presented (active drug vs. placebo), ^€^Difference between the baseline value and value obtained at the end of the trial, *Worsening nephropathy was defined as doubling of creatinine level, initiation of dialysis, renal transplantation, or creatinine .6.0 mg/dL (530 mmol/L) in SAVOR-TIMI 53; as the new onset of macroalbuminuria (urine albumin creatinine ratio.300mg/g) or a doubling of the serum creatinine level and an eGFR of ≤45 mL/min/1.73m^2^, the need for continuous renal-replacement therapy, or death from renal disease in LEADER, SUSTAIN-6, and EMPA-REG OUTCOME; and as 40% reduction in eGFR, renal-replacement therapy, or death from renal causes in CANVAS. Worsening nephropathy was a pre-specified exploratory adjudicated outcome in SAVOR-TIMI 53, LEADER, SUSTAIN-6, and CANVAS but not in EMPA-REG OUTCOME, **death due to renal failure, sustained decrease of at least 50% in eGFR (CARMELINA trial), ***calculated as mean ± standard deviation (95% CI).

**References**

1. Scirica BM, Bhatt DL, Braunwald E, Steg PG, Davidson J, Hirshberg B, et al. Saxagliptin and cardiovascular outcomes in patients with type 2 diabetes mellitus. N Engl J Med 2013;369:14-1317–26.
2. Mosenzon O, Leibowitz G, Bhatt DL, Cahn A, Hirshberg B, Wei C, et al. Effect of saxagliptin on renal outcomes in the SAVOR-TIMI 53 trial. Diabetes Care 2017;40:1-69–76.
3. McGuire DK, Alexander JH, Johansen OE, Perkovic V, Rosenstock J, Cooper ME, et al. Linagliptin effects on heart failure and related outcomes in individuals with type 2 diabetes mellitus at high cardiovascular and renal risk in CARMELINA. Circulation. 2019; 139:3-351-61.
4. White WB, Cannon CP, Heller SR, Nissen SE, Bergenstal RM, Bakris GL, et al. Alogliptin after acute coronary syndrome in patients with type 2 diabetes. N Engl J Med 2013;369:14-1327–35.
5. Green JB, Bethel MA, Armstrong PW, Buse JB, Engel SS, Garg J, et al. Effect of sitagliptin on cardiovascular outcomes in type 2 diabetes. N Engl J Med 2015;373:3-232–42.
6. Cornel JH, Bakris GL, Stevens SR, Alvarsson M, Bax WA, Chuang LM, et al. Effect of sitagliptin on kidney function and respective cardiovascular outcomes in type 2 diabetes: outcomes from TECOS. Diabetes Care 2016;39:12-2304–10.
7. Zinman B, Wanner C, Lachin JM, Fitchett D, Bluhmki E, Hantel S, et al. Empagliflozin, cardiovascular outcomes, and mortality in type 2 diabetes. N Engl J Med. 2015;373:22-2117-28.
8. Wanner C, Inzucchi SE, Lachin JM, Fitchett D, von Eynatten M, Mattheus M, et al. Empagliflozin and progression of kidney disease in type 2 diabetes. N Engl J Med 2016;375:4-323–34.
9. Cherney DZI, Zinman B, Inzucchi SE, Koitka-Weber A, Mattheus M, von Eynatten M, et al. Effects of empagliflozin on the urinary albumin-to-creatinine ratio in patients with type 2 diabetes and established cardiovascular disease: an exploratory analysis from the EMPA-REG OUTCOME randomised, placebo-controlled trial. Lancet Diabetes Endocrinol 2017;5:7-610–21.
10. Neal B, Perkovic V, Mahaffey KW, de Zeeuw D, Fulcher G, Erondu N, et al. Canagliflozin and cardiovascular and renal events in type 2 diabetes. N Engl J Med 2017;377:7-644–57.
11. Neal B, Perkovic V, Mahaffey KW, de Zeeuw D, Fulcher G, Erondu N, et al. Canagliflozin and cardiovascular and renal events in type 2 diabetes. N Engl J Med. 2017;377:7-644-57.
12. Wiviott SD, Raz I, Bonaca MP, Mosenzon O, Kato ET, Cahn A, et al. Dapagliflozin and cardiovascular outcomes in type 2 diabetes. N Engl J Med. 2019;380:4-347-57.
13. Marso SP, Daniels GH, Brown-Frandsen K, Kristensen P, Mann JF, Nauck MA, et al. Liraglutide and cardiovascular outcomes in type 2 diabetes. N Engl J Med 2016;375:4-311–22.
14. Mann JFE, Orsted DD, Brown-Frandsen K, Marso SP, Poulter NR, Rasmussen S, et al. Liraglutide and renal outcomes in type 2 diabetes. N Engl J Med 2017;377:9-839–48.
15. Marso SP, Bain SC, Consoli A, Eliaschewitz FG, Jodar E, Leiter LA, et al. Semaglutide and cardiovascular outcomes in patients with type 2 diabetes. N Engl J Med 2016;375:19-1834–44.
16. Pfeffer MA, Claggett B, Diaz R, Dickstein K, Gerstein HC, Køber LV, et al. Lixisenatide in patients with type 2 diabetes and acute coronary syndrome. N Engl J Med. 2015 Dec 3;373(23):2247-57.
17. Holman RR, Bethel MA, Mentz RJ, Thompson VP, Lokhnygina Y, Buse JB, et al. Effects of once-weekly exenatide on cardiovascular outcomes in type 2 diabetes. N Engl J Med 2017;377:13-1228–39.
18. Chen Y, Tarng D, Chen H. Renal Outcomes of Pioglitazone Compared with Acarbose in Diabetic Patients: A Randomized Controlled Study. PLoS One. 2016; 11(11): e0165750.
19. Kernan WN, Viscoli CM, Furie KL, Young LH, Inzucchi SE, Gorman M, et al. Pioglitazone after Ischemic Stroke or Transient Ischemic Attack. N Engl J Med. 2016;374:14-1321-31.
20. Gerstein HC, Bosch J, Dagenais GR, Díaz R, Jung H, Maggioni AP, et al. Basal insulin and cardiovascular and other outcomes in dysglycemia. N Engl J Med. 2012;367:4-319-28.
21. Marso SP, McGuire DK, Zinman B, Poulter NR, Emerson SS, Pieber TR, et al. Efficacy and Safety of Degludec versus Glargine in Type 2 Diabetes. N Engl J Med 2017;377:8-723-32.
22. King P, Peacock I, Donnelly R. The UK prospective diabetes study (UKPDS): clinical and therapeutic implications for type 2 diabetes. Br J Clin Pharmacol. 1999;48:5-643-8.
